# Parameters of Capsulorrhexis and Intraocular Lens Decentration After Femtosecond and Manual Capsulotomies in High Myopic Patients With Cataracts

**DOI:** 10.3389/fmed.2021.640269

**Published:** 2021-03-11

**Authors:** Yanan Zhu, Kexin Shi, Ke Yao, Yuyan Wang, Sifan Zheng, Wen Xu, Peiqing Chen, Yibo Yu, Xingchao Shentu

**Affiliations:** ^1^The Eye Center, Second Affiliated Hospital of School of Medicine, Zhejiang University, Hangzhou, China; ^2^Zhejiang Provincial Key Lab of Ophthalmology, Hangzhou, China; ^3^GKT School of Medical Education, King's College London, London, United Kingdom

**Keywords:** cataract, high myopia, intraocular lens, femtosecond laser capsulotomy, continuous curvilinear capsulorrhexis

## Abstract

**Purpose:** To compare the parameters of capsulorrhexis and intraocular lens decentration after femtosecond laser capsulotomy and manual continuous curvilinear capsulorrhexis in high myopic patients with cataracts.

**Methods:** This is a prospective consecutive non-randomized comparative cohort study. Selected patients with axial length > 26.0 mm were divided into femtosecond laser capsulotomy (FS) group and manual continuous curvilinear capsulorrhexis (CCC) group. Five experienced phacoemulsification surgeons conducted all surgeries. Intraoperative complications and post-operative anterior segment photography were recorded. Intraocular lens decentration, area of capsulorrhexis, circularity, and capsule overlap were measured at 1 week, 1 month, and 2 years after surgery. Between group differences of parameters were determined with independent-sample *t*-test or the Mann–Whitney *U*-test, analysis of variance test, Pearson chi-square test, and Spearman rank correlation test.

**Results:** The study included 142 eyes (108 patients), 68 eyes in the FS group, and 74 eyes in the CCC group. At 1 week, 1 month, and 2 years after surgery, the area of capsulorrhexis in the CCC group was significantly larger than in the FS group (*P* < 0.05), while no significant difference was noted in circularity values. The complete overlap ratio in the FS group was significantly higher than that in the CCC group (*P* < 0.05) at each measured timepoint. Significant correlations were noted between the anterior chamber depth and the area of capsulorrhexis in the CCC group (*R* = 0.25, *P* = 0.04), but did not correlate in the FS group (*P* > 0.05). In patients with an anterior chamber depth >3 mm, the capsule-intraocular lens (IOL) overlap of the CCC group was less than that of the FS group at all measured timepoints after surgery (*P* < 0.05). Meanwhile, the IOL decentration in the CCC group was significantly greater than that of the FS group in those patients at 2 years after surgery (*P* < 0.05).

**Conclusion:** In high myopic patients with cataracts, with anterior chamber depth more than 3 mm, femtosecond laser capsulotomy can achieve better capsulorrhexis sizing and centering. Due to more precise capsulotomy and a better capsule-IOL overlap in the FS group, femtosecond laser capsulotomy resulted in better long-term centration of the IOL.

## Introduction

High myopia is typically defined as refraction >-6 D or axial length >26.0 mm (1, 2). In the past few decades, the prevalence of high myopia has markedly increased. It is estimated the incidence of high myopia will increase to ~10% worldwide by 2050 (3). High myopia is one of the common causes of vision loss and can cause many complications, including cataracts (4). High myopic patients are likely to have an enlarged capsular bag with weak zonules and tend to develop cataracts earlier than emmetropic patients (5, 6). This increases the risk of post-operative intracapsular IOL dislocation. Cataract surgery combined with high myopia poses a greater challenge. Patients with high myopia have certain pathological changes, such as a deep anterior chamber, thin scleral wall, long axis that can lead to measurement errors, intraoperative fluctuations of anterior chamber, and/or changes of pupil size (7).

The effective position of intraocular lens is very important to the patient's visual quality after surgery. The architecture of the capsulorrheis greatly affects the position of lens, which in turn affects the subsequent refractive outcome. A perfectly circular and properly sized capsulorrhexis allows the capsular bag to completely envelop the IOL optic, providing a more predictable effective lens position and achieving optimal refractive outcome. However, if the capsulorrhexis is too large, the IOL optic can be tilted or decentered, resulting in astigmatism or compromised retinal image (8). At present, manual continuous curvilinear capsulorhexis (mainly used in conventional phacoemulsification surgery) has many uncertainties, especially in high myopia (9). In recent years, with the advent of femtosecond lasers in cataract surgery, a predictably sized, centered, and shaped anterior capsulotomy became possible (10). Earlier studies showed that femtosecond laser can achieve more regular circular capsulorrhexis (11, 12), which opens up a new opportunity for cataract surgery in patients with high myopia.

In this study, we used a large sample size in a prospective trial to compare femtosecond laser-assisted capsulotomy and manual capsulotomy in high myopic cases with cataracts with a 2-years follow-up. By measuring and comparing size and positioning parameters, the advantages of the two surgical methods were evaluated.

## Methods

### Patients

This study was approved by the Institutional Review Board of the Second Affiliated Hospital of the Zhejiang University School of Medicine in Hangzhou, China. All research and data collection practices adhered to the tenets of the Declaration of Helsinki. The study was registered with the Chinese Clinical Trial Registry^1^. Written informed consent was obtained from all patients after they received a full explanation of the study.

The study consecutively recruited Chinese high myopic patients (axial length > 26.0 mm) with cataracts. All patients were given the option to choose femtosecond laser capsulotomies or continuous curvilinear capsulorrhexis. All patients had implantation of intraocular lenses. Each patient underwent a complete ophthalmologic evaluation. Patients with previous ocular surgery, trauma, active ocular disease, poorly dilated pupils, or known zonular weakness were excluded from the study. The selected patients were divided into two groups: FS and CCC. The FS group received femtosecond laser-assisted cataract surgery, and the CCC group received conventional phacoemulsification cataract surgery.

### Surgical Technique

Every patient accepted the standard surgical procedure. All surgeries were performed by 5 experienced phacoemulsification surgeons (KY, WX, XS, PC, and YY). Each surgeon had performed more than 500 femtosecond laser-assisted cataract surgeries and 5,000 conventional phacoemulsification cataract surgeries. Before surgery, both groups achieved pupil dilation with an instillation of 0.5% tropicamide.

In the FS group, femtosecond laser was applied during the capsulotomy and lens fragmentation. Disposable interface contact lenses with suction rings (Softfit Patient Interface, Alcon LenSx, Inc.) were used for the corneal applanation. LenSx software (version 2.23, Alcon LenSx, Inc.) was used to create a 5.0 mm capsulotomy, and nuclear prefragmention was performed to obtain 6 pieces in a cross pattern.

In the CCC group, anterior capsules were treated conventionally [staining with trypan blue 0.06% under sodium hyaluronate 1.7% ophthalmic viscosurgical device (OVD) (Amvisc Plus, Bausch & Lomb, Inc.)]. Capsule forceps were used to complete a 5.0 mm continuous curvilinear capsulorrhexis.

In both groups, a 2.0 mm single-plane main incision and a 0.8 mm side-port corneal incision were made with a keratome. Phacoemulsification was performed using a standard stop-and-chop technique with longitudinal phacoemulsification system (Stellaris, Bausch & Lomb, Inc.). All IOLs were folded and implanted in the capsular bag with the aid of an injection cartridge through the corneal wound. After the IOL implantation, the viscoelastic material was removed from the anterior chamber and the capsular bag by irrigation/aspiration. All incisions were left sutureless. All patients received standard regimen consisting of topical dexamethasone tobramycin 4 times a day for 2 weeks and pranoprofen for 1 month after surgery.

### Patient Evaluation

**Figure 1:**
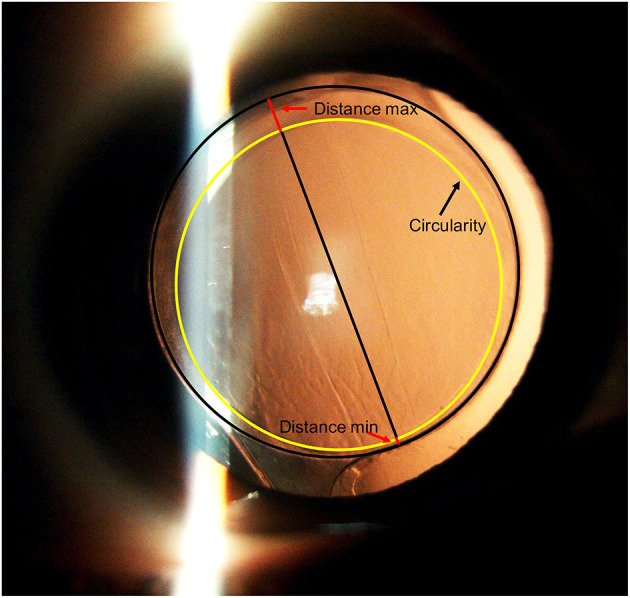
Post-operative anterior segment photography with dilated pupil. The black circle shows the edge of the IOL, the yellow line shows the edge of the capsulorhexis. The red lines indicate the shortest and longest distance between the edge of the capsulorrhexis and the IOL optic edge along an elongated radius of the capsulorrhexis(distance min, distance max).

**Figure 2:**
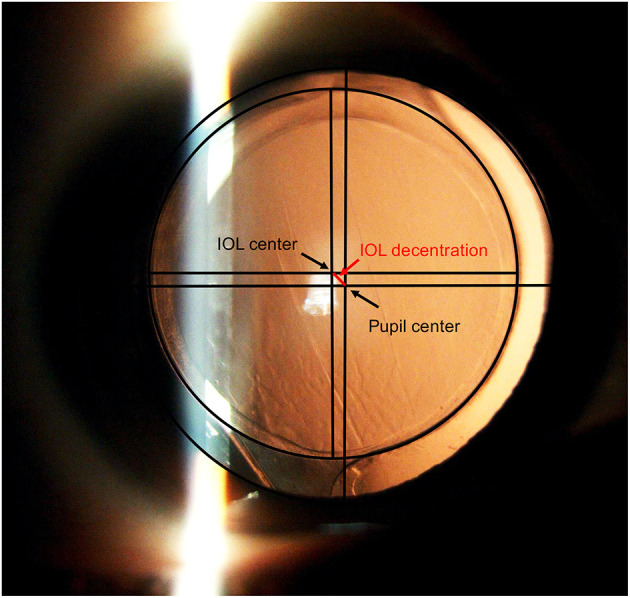
Post-operative anterior segment photography with dilated pupil. The black circle inside shows the edge of the IOL, the black circle outside shows the edge of the pupil. The black lines indicate the horizontal and vertical diameters of the pupil and IOL. The red line between the pupil center and IOL center indicates the IOL decentration.

Preoperatively, the medical histories of all patients were recorded. Comprehensive evaluations were also performed, including an A-scan standardized ultrasound (US) (Cinescan, Quantel Medical SA) and an IOLMaster biometry (Carl Zeiss). Anterior segment photography with a dilated pupil was captured at 1 week, 1 month, and 2 years after surgery. Photographs were imported into AutoCAD 2018 image-processing for Windows software (version 22.0, AutoDesk) to measure the IOL decentration and the following capsulotomy parameters: area of the capsulorrhexis, circularity, and the shortest and longest distance between the edge of the capsulorrhexis, and the IOL optic edge (distance min, distance max) along an elongated radius of the capsulorrhexis (Figure 1). Circularity is a parameter used for determining the regularity of capsulotomy shape according to the following formula: circularity = 4π(area/perimeter^2^). The quotient of the shortest and longest distance between the edge of the capsulorrhexis and the edge of the IOL optic was calculated to determine capsule-IOL overlap (capsule−IOL overlap = distance min/distance max). Circularity and overlap values of 1.0 indicate a perfect circle and an absolute regularly overlapping anterior capsule on the optic of the implanted IOL, respectively. Complete overlap is defined as when the edge of the capsulorhexis is completely within the IOL edge. If a part of capsulohexis edge outside the IOL edge, it is regarded as an incomplete overlap. AutoCAD 2018 gives a vector (determined by its length and angle to the horizontal plane) between the pupil center and center of the IOL (Figure 2). IOL decentration is the vector length between these 2 centers. The diameter of the implanted IOL was used as a scale to eliminate the magnification effect of the cornea. All measurements were taken by the same technician (who was masked to the patients) and conditions were kept consistent for all eyes operated.

### Statistical Analysis

The sample size was determined based on a power calculation (power 0.85; *P* = 0.05) using standard deviations obtained in our pre-study (13). At least 49 patients per group were required to be included in the analysis to achieve sufficient power in the statistical calculations.

Categorical data were defined as the number and percentage, and A Pearson chi-square analysis was used for statistical analysis. Continuous variables were defined as means ± SD, and between-group comparative statistics were determined using the independent-sample *t*-test or the Mann–Whitney *U*-test, depending on the departure from normal distribution. The difference of capsulorrhexis parameters among multiple groups was analyzed by one-way analysis of variance test. Correlations between parameters were analyzed with Spearman rank correlation test. A *P* < 0.05 was considered statistically significant. All analyses (except when noted) were performed using IBM SPSS Statistics software (version 26.0, IBM Corp).

## Results

A total of 142 eyes (108 patients) attended at least 2 follow-up visits. Missing data were due to personal inconvenience, refusal to mydriasis, or temporary device failure. There were 74 eyes in the FS group and 68 eyes in the CCC group. No statistically significant differences were noted between the CCC group and FS groups in regards to age, gender distribution, axial length, or anterior chamber depth (Table 1). The mean age was 64 ± 11 years in the CCC group and 61 ± 13 years in the FS Group (*P* = 0.26). There were 45 women (66.2%) in the CCC group and 49 women (66.2%) in the FS group (*P* = 1.00). Axial length was 29.53 ± 2.40 mm in the CCC group and 29.05 ± 2.17 mm in the FS group (*P* = 0.21). Anterior chamber depth was 2.89 ± 0.68 mm in the CCC group and 2.77 ± 0.69 mm in the FS group, separately (*P* = 0.30). Table 2 shows that each surgeon performed a similar number of femtosecond laser capsulotomies and manual capsulotomies. There was no significant difference of parameters in capsulotomies between the surgeons (*P* > 0.05).

**Table 1:** Demographics of patients who underwent manual continuous curvilinear capsulorrhexis or femtosecond laser capsulotomy.

**Demographic**	**CCC group**	**FS group**	***P*-value**
Mean age (y)	64 ± 11	61 ± 13	0.26
Sex (M:F)	23:45	25:49	1.00
Axial length (mm)	29.53 ± 2.40	29.05 ± 2.17	0.21
Anterior chamber depth (mm)	2.89 ± 0.68	2.77 ± 0.69	0.30

**Table 2:** Statistical information about the number of operations performed by surgeons and parameter of capsulotomies after surgery.

	**Number of operations**		**Area of capsulorrhexis (mm**^****2****^**)**			
**Surgeon**	**CCC**	**FS**	**CCC**	***P*-value**	**FS**	***P*-value**
Ke Yao	16	18	22.25 ± 1.94	0.68	21.08 ± 1.42	0.41
Wen Xu	14	16	22.11 ± 2.62		21.28 ± 1.76	
Xingchao Shentu	15	14	21.34 ± 1.44		20.79 ± 1.90	
Peiqing Chen	12	14	21.29 ± 2.45		21.36 ± 1.18	
Yibo Yu	11	12	22.27 ± 2.52		20.77 ± 1.56	

Table 3 shows the parameters of the capsulotomies and IOL decentrations in the two study groups measured by AutoCAD. At 1 week, 1 month, and 2 years after surgery, the area of capsulorrhexis in the CCC group were significantly larger than those in the FS group (*P* < 0.05), while no significant difference was noted in circularity values. The complete overlap ratio in the FS group was significantly higher than that in the CCC group (*P* < 0.05) at each measured time point after surgery. However, in the cases with complete overlap, there was no significant difference in the capsule-IOL overlap between CCC group and FS group at all measured timepoints (*P* > 0.05). No significant difference was also noted in the IOL decentration between the two groups at all measured timepoints (*P* > 0.05).

**Table 3:** Parameters of capsulotomies and intraocular decentrations in eyes that underwent continuous curvilinear capsulorrhexis or femtosecond laser capsulotomy.

	**1 week**			**1 month**			**2 years**		
**Parameters**	**CCC**	**FS**	***P*-value**	**CCC**	**FS**	***P*-value**	**CCC**	**FS**	***P*-value**
Area of capsulorrhexis (mm^2^)	21.85 ± 2.18	21.07 ± 1.54^*^	0.01	21.02 ± 2.16	20.19 ± 2.03^*^	0.04	20.86 ± 2.22	19.76 ± 2.17^*^	0.01
Circularity	0.99 ± 0.02	0.99 ± 0.01	0.06	0.99 ± 0.01	0.99 ± 0.01	0.13	0.99 ± 0.02	0.99 ± 0.03	0.89
Complete overlap (%)	94	100^*^	0.03	93	100^*^	0.02	90	99^*^	0.02
Capsule-IOL overlap	0.41 ± 0.19	0.44 ± 0.17	0.23	0.40 ± 0.19	0.45 ± 0.16	0.10	0.41 ± 0.19	0.47 ± 0.17	0.06
IOL decentration (mm)	0.12 ± 0.16	0.12 ± 0.14	0.98	0.17 ± 0.16	0.16 ± 0.16	0.73	0.23 ± 0.17	0.20 ± 0.16	0.22

* *P < 0.05 between groups at the given time point using repeated measures analysis of variance*.

**Figure 3:**
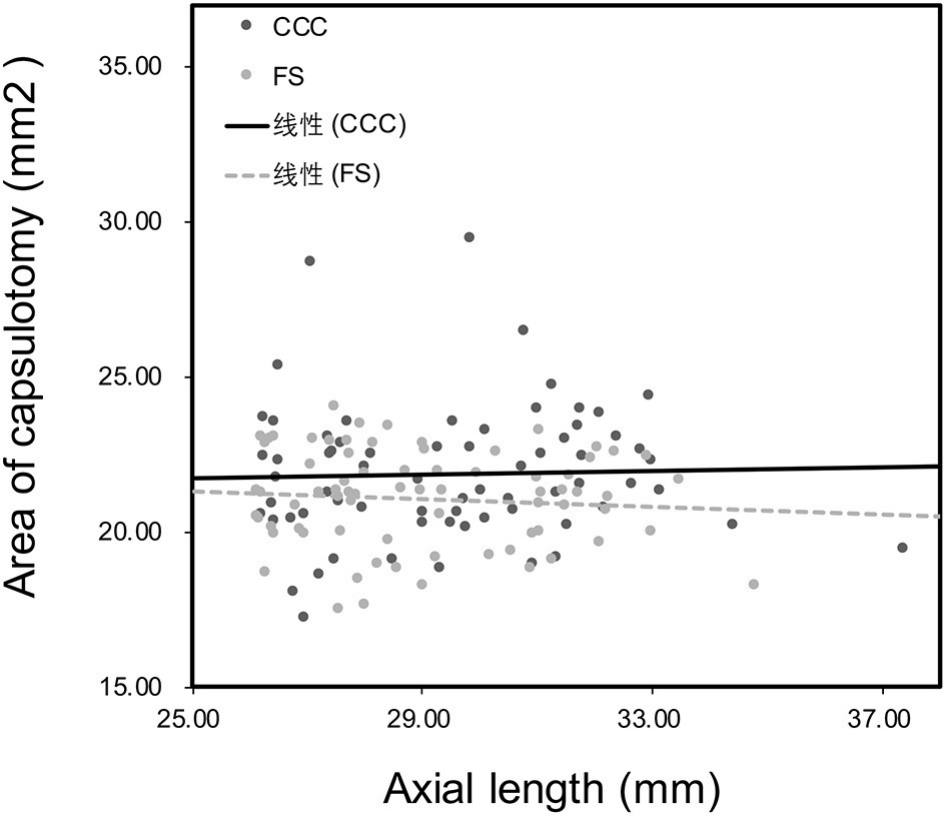
Correlation between axial length and area of capsulotomy 1 week after surgery. No correlation was found in the manual capsulotomy group (CCC) (*R* = 0.02, *P* = 0.88) and laser capsulotomy group (FS) (*R* = −0.08, *P* = 0.48).

**Figure 4:**
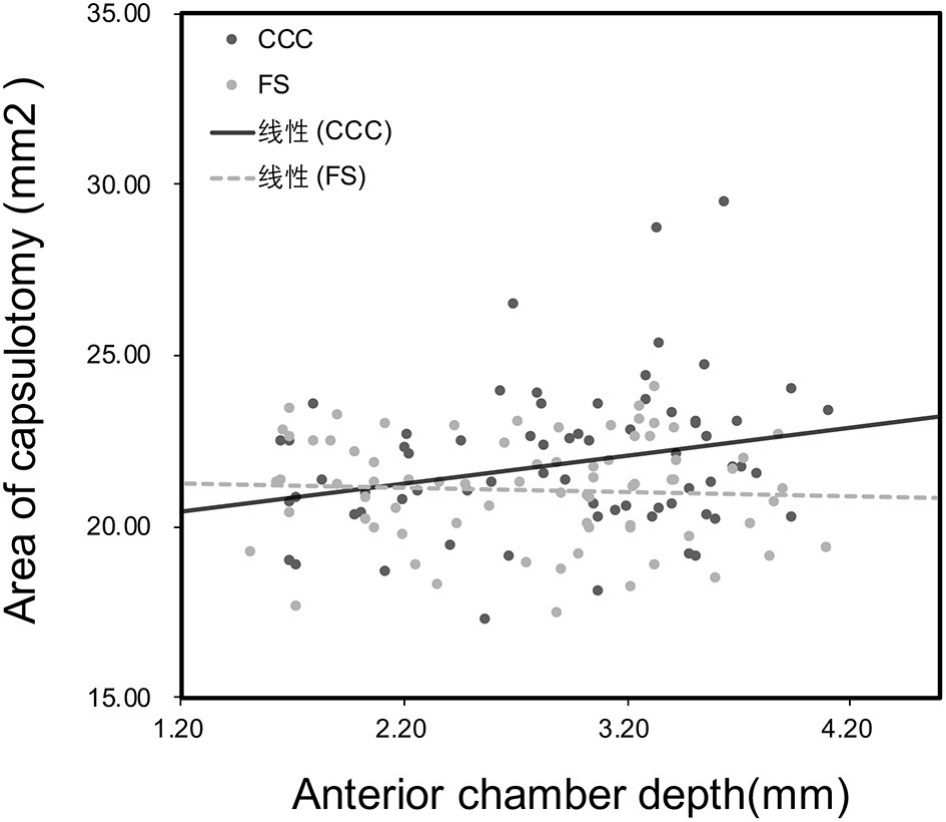
Correlation between anterior chamber depth and area of capsulotomy 1 week after surgery. The correlation was significant in the manual capsulotomy group (CCC) (*R* = 0.25, *P* = 0.04) but not in the laser capsulotomy group (FS) (*R* = −0.04, *P* = 0.75).

Figure 3 shows no significant correlation was found between the axial length and the area of capsulotomy in either study group (*P* > 0.05). There was a statistically significant correlation between anterior chamber depth and area of capsulotomy in the CCC group (*R* = 0.25, *P* = 0.04), however no statistically significant correlation was noted between these parameters in the FS group (*P* > 0.05; Figure 4). Patients were subsequentoy divided into the normal anterior chamber group (anterior chamber depth <3.00 mm) and deep anterior chamber group (anterior chamber depth ≥3.00 mm), according to the results of the correlation analysis.

There was no statistical difference in age, gender distribution, axial length or anterior chamber depth in the sub-group analysis of anterior chamber depth ≥3 mm or <3 mm in both the CCC group and the FS group (Table 4). Among patients with normal anterior chambers, the CCC group had 33 eyes and the FS group had 40 eyes. We found that in patients with normal anterior chamber, there were no significant differences in the parameters of the capsulorrhexis and the IOL decentration between the FS group and the CCC group (Table 5). In patients with deep anterior chamber, there were 35 eyes in the CCC group and 34 eyes in the FS group. Many statistically significant differences were noted between the two groups. As shown in Table 6, the area of capsulorrhexis of the CCC group was significantly larger than that of the FS group at all measured timepoints after surgery (*P* < 0.05). The complete overlap ratio in the FS group was also significantly higher than that in the CCC group (*P* < 0.05) at 1 month and 2 years after surgery. The capsule-IOL overlap of the CCC group was less than the FS group at each timepoint after surgery (*p* < 0.05) and the IOL decentration in the CCC group was significantly higher than the FS group at 2 years after surgery (*P* < 0.05).

**Table 4:** Demographics of patients (anterior chamber depth <3.00 mm and anterior chamber depth ≥ 3.00 mm) who underwent manual continuous curvilinear capsulorrhexis or femtosecond laser capsulotomy.

	**Demographic**	**CCC group**	**FS group**	***P*-value**
Anterior chamber depth <3.00 mm	Mean age (y)	64 ± 12	61 ± 14	0.49
	Sex (M:F)	12:21	14:26	0.90
	Axial length (mm)	29.67 ± 2.48	28.69 ± 2.10	0.07
	Anterior chamber depth (mm)	2.32 ± 0.44	2.25 ± 0.45	0.48
Anterior chamber depth ≥3.00 mm	Mean age (y)	64 ± 9	64 ± 12	0.81
	Sex (M:F)	11:24	11:23	0.93
	Axial length (mm)	29.40 ± 2.35	29.48 ± 2.21	0.89
	Anterior chamber depth (mm)	3.43 ± 0.33	3.39 ± 0.30	0.62

**Table 5:** Parameters of capsulotomies and intraocular decentrations in eyes that underwent continuous curvilinear capsulorrhexis or femtosecond laser capsulotomy (anterior chamber depth <3.00 mm).

	**1 week**			**1 month**			**2 years**		
**Parameters**	**CCC**	**FS**	***P*-value**	**CCC**	**FS**	***P*-value**	**CCC**	**FS**	***P*-value**
Area of capsulorrhexis (mm^2^)	21.49 ± 1.85	21.00 ± 1.60	0.22	20.82 ± 2.14	20.34 ± 2.34	0.43	20.66 ± 1.89	19.85 ± 2.21	0.14
Circularity	0.99 ± 0.02	0.99 ± 0.01	0.31	0.99 ± 0.02	0.99 ± 0.01	0.14	0.99 ± 0.02	0.99 ± 0.03	0.94
Complete overlap (%)	97	100	0.27	97	100	0.27	91	98	0.22
Capsule-IOL overlap	0.43 ± 0.21	0.41 ± 0.18	0.67	0.42 ± 0.19	0.43 ± 0.16	0.85	0.42 ± 0.18	0.44 ± 0.15	0.68
IOL decentration (mm)	0.14 ± 0.18	0.11 ± 0.14	0.47	0.17 ± 0.18	0.15 ± 0.16	0.71	0.20 ± 0.19	0.20 ± 0.17	0.91

*P < 0.05 between groups at the given time point using repeated measures analysis of variance.

**Table 6:** Parameters of capsulotomies and intraocular decentrations in eyes that underwent continuous curvilinear capsulorrhexis or femtosecond laser capsulotomy (anterior chamber depth ≥3.00 mm).

	**1 week**			**1 month**			**2 years**		
**Parameters**	**CCC**	**FS**	***P*-value**	**CCC**	**FS**	***P*-value**	**CCC**	**FS**	***P*-value**
Area of capsulorrhexis (mm^2^)	22.19 ± 2.43	21.16 ± 1.49^*^	0.04	21.18 ± 2.21	20.02 ± 1.63^*^	0.03	21.04 ± 2.50	19.64 ± 2.15^*^	0.03
Circularity	0.99 ± 0.01	0.99 ± 0.01	0.06	0.99 ± 0.01	0.99 ± 0.01	0.51	0.99 ± 0.01	0.99 ± 0.01	0.60
Complete Overlap (%)	91	100	0.08	89	100^*^	0.04	89	100^*^	0.04
Capsule-IOL Overlap	0.38 ± 0.17	0.48 ± 0.15^*^	0.01	0.37 ± 0.20	0.48 ± 0.15^*^	0.04	0.40 ± 0.20	0.51 ± 0.17^*^	0.03
IOL Decentration (mm)	0.10 ± 0.14	0.13 ± 0.14	0.38	0.17 ± 0.15	0.16 ± 0.16	0.93	0.27 ± 0.19	0.19 ± 0.15^*^	0.04

* *P < 0.05 between groups at the given time point using repeated measures analysis of variance*.

In addition, in both the CCC and the FS group, no cases of capsular contraction syndrome was found. The area of the capsulorrhexis at 1 month and 2 years after surgery were significantly smaller than the capsular opening area at 1 week after surgery (*P* < 0.05) in both gruops. However, there was no significant difference between the value of the reduced capsulorrhexis area of the CCC group and the FS group measured at 1 week-1 month and 1 week-2 years (1.44 ± 1.31 vs. 1.75 ± 1.45 mm^2^, *P* = 0.53; 1.27 ± 1.46 vs. 1.98 ± 1.84 mm^2^, *P* = 0.47; respectively).

## Discussion

Femtosecond laser, a new technology, has been applied in cataract surgery in recent years. Previous studies had reported that femtosecond laser capsulotomy improve the centration, circularity and precision of anterior capsulorrhexis (14, 15). The size and shape of the anterior capsulorrhexis greatly affect surgical outcomes, including the position of the lens and the subsequent refractive outcomes. If the capsulorrhexis is too large, the IOL may be decentered, resulting in visual dysfunction such as refractive error and high-order aberrations increasing (16). If it is too small, the capsular bag is highly likely to contract and cause complications, such as IOL loop curling and decentration (17). High myopia is the most common risk factor for advanced intracapsular IOL dislocation (18). A previous study had also demonstrated that the frequency of IOL tilt and decentration was significantly higher in cataract eyes with high myopia than that in non-myopia cataract eyes (19). Due to the special pathological changes of high myopia, a perfectly circular and properly sized capsulorrhexis is extremely important.

Surgeons have applied femtosecond lasers to highly myopic cataracts. Previous studies indicated that femtosecond laser capsulotomy, compared with manual capsulotomy, has a more regular shape of capsulorrhexis, a higher capsule-IOL overlap, and a better IOL centration in myopic eyes (20, 21). It was also reported that the size of capsulorrhexis area and the IOL decentration in manual capsulotomy were positively correlated with the axial length, while femtosecond laser capsulotomy eliminated these errors (20). However, femtosecond laser and highly myopic cataracts were rarely reported, with a relatively small sample size and a relatively short follow-up time.Therefore, in order to further investigate the effect of femtosecond laser application in highly myopic catarats, we conducted a prospective large-sample long-term study.

In our study, we evaluated the capsulorrhexis size, circularity, IOL decentration, and capsule-IOL overlap in high myopic patients with cataracts. In general, we found that femtosecond laser capsulotomy has better parameters of capsulorrhexis and caspule-IOL overlap, which included the capsulorrhexis area being more precise and the complete overlap ratio being superior to compared manual capsulotomy. These results are all supported by previous studies (20) and are consistent with those seen in common cataracts (22, 23). However, there is no significant difference in the circularity of the 2 types of capsulotomy in this study. Due to the advanced operation technology, the circularity of both surgical methods is extremely high (above 99%), compared with the circularity in an earlier study (20, 22) (~85%). The circularity of the capsulorrhexis much depends on the surgeon's skill.

We attempted to find the characteristics of highly myopic cataracts. In this study, we investigated the correlation between parameters of the capsulorrxis and the axial length at first. Our results contradicted the results from Nagy et al. and no significant correlation was found between parameters of the capsulorrxis and the axial length. As the angle and depth of the capsulotomic forceps manipulation mainly depend on the anterior chamber depth, we studied the correlation between the anterior chamber depth and the capsulorrxis parameters. Our study showed a significant positive correlation between the two in the CCC group, but no correlation in the FS group. The relationship between anterior chamber depth and axial length in high myopia is controversial. Previous studies had demonstrated that anterior chamber depth is positively correlated with axial length in myopic eyes (24). However, evidence has also emerged that the increase of axial length in patients with long eye axis was mainly due to the expansion and lengthening of vitreous cavity, rather than the change of anterior segment morphology. In eyes with long axial length, the correlation between anterior chamber depth and axial length disappeared (25). Our study has shown compared with the axial length, the depth of the anterior chamber has a greater impact on the operation of high myopic cataract surgery. We can conclude that the capsulorrhexis parameter is related to depth of the anterior chamber rather than axial length.

Our study found when the anterior chamber depth was <3 mm, the manual capsulorrhexis was significantly larger than femtosecond and more eccentric. Previous studies have also suggested statistically significant correlations between the anterior chamber depth and the pupil diameter, as well as the white-to-white corneal diameter in myopia (26, 27). Our study indicated that femtosecond laser capsulotomy has absolute advantages when the anterior chamber depth was >3 mm. This is due to the significant difference in the pupil reference and the operating angle, which causes the difficulty in manual capsulotomy. When the anterior chamber depth was <3 mm, experienced surgeons can perform capsulotomy adequately according to their experience. The long-term centration of the IOL depends on the size and location of the capsulorrhexis and the extent of the IOL coverage (22). We further found that when the anterior chamber depth was >3 mm, the FS group had better IOL centration at 2 years after surgery due to a better capsule-IOL overlap and more precise capsulorrhexis area. Because of the long-term centration of the IOL, patients can have better visual quality. Some studies had indicated that femtosecond laser surgery can provide patients with better visual quality after premium IOL implanation, such as the toric IOL and multifocal IOL (28, 29).

High myopia, small capsulorrhexis, and hydrophilic IOL are all considered risk factors for anterior capsular contraction (19, 30). In this study, although the diameter of the FS group's capsulorrhexis was smaller than the CCC group, we did not find any cases of capsular contraction syndrome. The area of the capsulorrhexis of the two groups was reduced to a certain extent after surgery, but there was no significant difference in the value of the reduced area in the two groups. Therefore, femtosecond laser does not increase the risk of capsular contraction in high myopic patients with cataracts. not only is femtosecond laser effective and comparatively safe in cataract surgery for high myopia.

Our main limitation concerns evaluating the IOL decentation. Only the value on the horizontal plane of the IOL was measured, and the changes in the anteroposterior positions of the IOL were not considered. It would be pertient to examine whether the effect of capsulotomies influences the tilting of the IOL over time. In addition, due to the additional cost of using femtosecond lasers, we must respect the wishes of patients to choose surgery. This limits the randomization of the study. A previous study also concluded that thicker lens contribute to greater IOL decentration (8). Due to the lack of equipment to measure lens thickness at the beginning of our study, this factor was not explored. Whether the percentage and magnitude of IOL decentration are different between the cases with complete or incomplete overlap should also be explored. Unforunately, the number of cases with incomplete overlap was too small for statistical comparison.

In conclusion, in high myopic patients with cataracts and anterior chamber depth >3 mm, femtosecond laser capsulotomy is the more ideal choice for surgery because it has a better IOL overlap and a better IOL positioning.

## Data Availability Statement

The raw data supporting the conclusions of this article will be made available by the authors, without undue reservation.

## Ethics Statement

The studies involving human participants were reviewed and approved by Ethics Committee of Second Affiliated Hospital of Zhejiang University, College of Medicine. The patients/participants provided their written informed consent to participate in this study. Written informed consent was obtained from the individual(s) for the publication of any potentially identifiable images or data included in this article.

## Author Contributions

XS: study concept and design. KY, XS, WX, PC, YY, and YW: data collection. YZ, KS, and XS: analysis and interpretation of data. YZ, KS, XS, and SZ: drafting and critical revision of the manuscript. All authors contributed to the article and approved the submitted version.

## Conflict of Interest

The authors declare that the research was conducted in the absence of any commercial or financial relationships that could be construed as a potential conflict of interest.

## Footnotes

^1^Chinese Clinical Trial Registry. *Clinical Outcomes and Complications of Femtosecond Laser-Assisted Cataract Surgery Versus Conventional Phacoemulsification Surgery*. ChiCTR-ONN-17010319. Available online at: http://www.chictr.org.cn/showproj.aspx?proj=17486 (accessed January 20, 2021).
